# Process attributes in bio-ontologies

**DOI:** 10.1186/1471-2105-13-217

**Published:** 2012-08-28

**Authors:** André Q Andrade, Ward Blondé, Janna Hastings, Stefan Schulz

**Affiliations:** 1 Institute for Medical Informatics, Statistics and Documentation, Medical University of Graz, Graz, Austria; 2 School of Information Science, Federal University of Minas Gerais, Belo Horizonte, Brazil; 3 Cheminformatics and Metabolism, European Bioinformatics Institute, Hinxton, UK; 4 Swiss Centre for Affective Sciences, University of Geneva, Geneva, Switzerland; 5 Institute for Medical Biometry and Medical Informatics, University Medical Center, Freiburg, Germany

## Abstract

**Background:**

Biomedical processes can provide essential information about the (mal-) functioning of an organism and are thus frequently represented in biomedical terminologies and ontologies, including the GO Biological Process branch. These processes often need to be described and categorised in terms of their attributes, such as rates or regularities. The adequate representation of such process attributes has been a contentious issue in bio-ontologies recently; and domain ontologies have correspondingly developed *ad hoc* workarounds that compromise interoperability and logical consistency.

**Results:**

We present a design pattern for the representation of process attributes that is compatible with upper ontology frameworks such as BFO and BioTop. Our solution rests on two key tenets: firstly, that many of the sorts of process attributes which are biomedically interesting can be characterised by the ways that repeated parts of such processes constitute, in combination, an overall process; secondly, that entities for which a full logical definition can be assigned do not need to be treated as primitive within a formal ontology framework. We apply this approach to the challenge of modelling and automatically classifying examples of normal and abnormal rates and patterns of heart beating processes, and discuss the expressivity required in the underlying ontology representation language. We provide full definitions for process attributes at increasing levels of domain complexity.

**Conclusions:**

We show that a logical definition of process attributes is feasible, though limited by the expressivity of DL languages so that the creation of primitives is still necessary. This finding may endorse current formal upper-ontology frameworks as a way of ensuring consistency, interoperability and clarity.

## Background

While a static description of the structure of an organism can provide some information about the state of its functioning or malfunctioning at a given point in time, the adequate description of its dynamic processes conveys a wealth of additional information. Biological processes include intracellular transformations, which consume and metabolise nutrients in order to produce energy, the overarching developmental process of a growing organism, the cycles of sleep and waking that structure our daily lives, and the pathological processes of unrestrained cellular proliferation characteristic for cancer. Many of the normal biological processes can be perturbed by pathological conditions. The understanding of their normal operation and the recognition of various sorts of processual pathologies such as delays or irregularities are essential to feed knowledge-based information systems that support data-driven biological research. The distinction between a static description of a biological structure on the one hand and the dynamic description of organismal processes on the other hand corresponds to the distinction between the Gene Ontology [1] branches for *cellular components* and *biological processes.* This points at a fundamental upper-level ontological division such as between entities that unfold through time and do not exist in full until they are completed (occurrents, such as processes) and entities that exist in full in every instant that they exist (continuants, such as objects) in the Basic Formal Ontology (BFO) [2]. Medical terminology systems similarly separate anatomical terms, e.g. "heart", from process terms such as such as "heartbeat", thus supporting proper and unambiguous definitions of pathological phenomena and clinical findings like regular heartbeats and arrhythmia, and for automatically distinguishing between normal and raised heart rates. Health-related processes and the attributes by which these phenomena are described and modified (e.g. heart beating, regular or arrhythmic), are common in health records and appear in bio-ontologies, such as the Gene Ontology and the Human Phenotype Ontology [3], as well as medical terminologies such as SNOMED CT.

However, the adequate ontological representation of attributes that modify processes such as rates and regularities – often referred to as process “qualities” – has been a contentious topic in recent years. Due to the lack of a proper and sound definition following the methodology of Ontological Realism [4] (as discussed below), “process qualities” were not included in previous BFO versions, where the category Quality is strictly limited to dependent entities that inhere in continuants.. Where ontologies aligned with BFO have needed to include such attributes, they have heretofore resorted to using ad-hoc workarounds, including representing the attributes of processes as if they were attributes of the participants, or representing complicated process hierarchies directly without reference to such attributes, having the consequence that there is no mechanism for comparison of processes based on their attributes.

Here, we present a novel solution to part of the problem of representing process attributes, based on two key tenets: firstly, that many of the sorts of process attributes of biomedical interest can be characterised by the ways that repeated parts of such processes combine to constitute the overall process; and secondly, that full logical definition of process attributes that follow this pattern is in principle possible, so that they do not need to be treated as primitives within ontologies. Using the Web Ontology Language (OWL) version 2 [5], we will show how full logical definitions can be created for representation of attributes relevant for modelling normal and abnormal heart rates and heart cycles. We will further discuss examples of process attributes for which the expressivity offered by OWL cannot fully formalise the attributes of processes, which could however be done by full first-order logic (FOL). We will evaluate these representations against a set of domain questions and tasks, as well as against a set of ontology evaluation criteria. Our solution is only applicable to process attributes which can be characterised in terms of parts of processes. Our use case for elucidation focuses on cyclic processes, but in our Discussion we additionally present some relevant examples of applicable non-cyclic processes.

The remainder of this paper is organised as follows. In the following sections we introduce the ontological and biomedical context. In the Results section, we present our sample ontologies together with their evaluation against the assigned evaluation criteria. Our Discussion provides a broader perspective, comparing our approach to alternative solutions that address the same issue, highlighting the limitations of our approach, and considering its relevance to alternative applications. Finally, we give our conclusions.

### Ontological realism and the challenge with process “qualities”

Ontological realism has been repeatedly advocated as a theory which is particularly useful for the development of scientific ontologies. It advocates adherence to a set of philosophically grounded tenets in order to improve the quality and interoperability of the resulting ontology artefacts [4]. One such tenet is strict alignment with upper-level ontologies. BFO [2], which most deeply implements the precepts of ontological realism, makes a fundamental distinction between types of entities based on the relationship to time: continuants are those entities that *continue* to exist through time, and exist in full at all times during which they exist, such as a human being, while occurrents are those entities that *unfold* or *happen* in time, and have temporal parts, such as the life of a human being. The biological and biomedical reality that is described by bio-ontologies such as those gathered by the OBO Foundry [6] is broadly divided between continuants such as cells, molecules, genes, and tissues, and the occurrents in which those continuants participate such as biological processes, chemical reactions. Continuants are further categorised in independent continuants, which do not depend on other entities for their existence, and dependent continuants, which require an independent continuant to inhere in and to be borne by. In BFO, qualities are special types of dependent continuants, for example, the *colour* of a fruit or the *weight* of a person; neither the colour nor the weight can exist without their respective bearers – the fruit and the person – existing.

Whereas other top level ontologies like DOLCE, GFO, or BioTop refrain from an upper level bipartition, in BFO, qualities are continuants and have therefore no temporal parts. As a consequence they cannot inhere in occurrents, which are, by definition, unfolding through time. If qualities could inhere in processes, then they would necessarily have temporal parts. For instance, assume that *being chronic* is a quality of a disease course process. It is not possible to make any statement on whether a process is chronic by inspecting a snapshot of this process at a particular moment in time. In contrast, it is perfectly possible to ascribe a colour to an apple at a moment in time. Furthermore, within the BFO framework, there is not even the possibility that there could be other sorts of quality-like entities that inhere in occurrents, which would correspond to attributes of processes: Qualities are the sorts of things that can change in their bearers over time (as an apple changes colour as it ripens), while processes cannot change over time, since processes *are* changes [7]. Each process has at least one (continuant) process participant and exactly one duration (the extent of the time interval between inception and ending). Processes can generally be split into numerous sub-processes, each of which having duration and some participant. Since many words used in natural language to qualify processes are actually time-related properties of the sub-events and their material participants, our working hypothesis is the following:

Considering the many interesting biological processes that are characterised by their duration, their parts (sub-processes), their participants, and the qualities of their participants, these parameters are sufficient for logically representing the meaning of the terms referring to the alleged process qualities.

However, practically all processes of interest within biomedical science, and therefore which are subject to descriptions and formalisations in biomedical ontologies, are highly complex entities, composed by numerous sub-processes of different kinds. Scientists are accustomed to using natural language for the assertion of biological events, in which adverbs modify verbs following the same pattern in which adjectives modify nouns, and considering that verbs frequently denote processes, users expect that adverbs should denote process attributes. Indeed, the use of process modifiers is widespread in the scientific literature. To represent these terms, several biomedical ontologies contain terms to modify processes, closely related to their use in natural language. In SNOMED CT [8], among the so-called qualifier values there are numerous that can be post-coordinated with disease concepts, such as *Deterioration of statusImprovement of statusChronic persistentProgressivePrecipitant* and many more. PATO [9], the ontology of phenotypic qualities, distinguishes numerous flavours of decreased and increased properties of processes, such as *occurrenceratefrequency*, and *duration*. Other properties include *synchronicityacceleration* (a property of change), *intensity*, and *regularity* / *normality* vs. *irregularity* / *abnormality* (regarding rhythm or sleep pattern), or having extra or missing sub-process parts. The Human Phenotype Ontology [3] does not separate the properties from their bearers, but contains equally numerous process terms with modifiers such as *Growth retardationSlowly progressive disorderAsymmetric growthParoxysmal bursts of laughter*, or *Limited shoulder movement*. Irregular or abnormal patterns of e.g. growth, movements etc. are also frequent.

Other ontologies pursue a different strategy. Instead of conceding that processes require modifiers, these ontologies ascribe the qualities to the participants of the described process. For instance, the Vital Sign Ontology (VSO) [10], an extension of the Ontology for General Medical Science (OGMS) [11] (itself an extension of the Basic Formal Ontology), also describes aspects commonly regarded as process modifiers, such as rates and modifiers – decreased and increased - drawn from PATO. In the VSO, a pulse rate is described as “The rate at which an artery pulses (i.e., participates in expansion-contraction cycles) as blood passes through it.”, and is represented as a quality of some artery. As a quality, it is something “that exists in full at any time in which it exists at all, persists through time while maintaining its identity and has no temporal parts”. In this paper we will present an alternative interpretation, considering that a pulse rate at t1 may have two different values depending on the duration of the measuring process. This interpretation, we argue, also allows representation of other clinically useful measures, such as rhythm, without compromising ontological soundness of the model.

We will demonstrate in what follows how the alleged “qualities of processes” can be fully defined in terms of patterns of relationships between the sub-processes and the overall containing process. Though adherent to BFO, we here define ‘process attribute’ as a defined class of occurrents that describes a process if its sub-processes and participants in a given time fit a given heuristically useful pattern, without committing (at this point) the class to the existence of any entity in reality. This provides a solution to the dilemma of whether to include them in bio-ontologies, since entities that are fully logically defined in terms of other, “genuine” entities, do not need to correspond to universals, and therefore preserve the ontological commitments of extant upper level ontologies. Once a clear consensus over a comprehensive approach to ontologically defining these attributes of processes is achieved, the definition of the ‘process attribute’ and its place in the upper-ontology hierarchy must be updated.

## Methods

### Use case: the heart cycle

Definitions for quality-like entities of processes depend on the domain of discourse. As a use case, we will assess to what extent we can provide ontological descriptions for a basic aspect of the heart physiology: the heart cycle and associated rates. As was shown in [12], representing heart rates is not trivial and requires further understanding of the rationale behind some medical statements. The main function of a heart is to pump blood to other organs. Since the process that realises this function is composed of mechanical movements of the heart, we can describe it by a series of muscle contraction and relaxation cycles. During the relaxation phase, called diastole, the blood fills the heart cavities, whereas in the contraction part, called systole, the blood is pumped from the heart ventricles in the peripheral circulatory system. A heart cycle is, therefore, composed of two distinct parts, each being a precondition for the next (that is, there can be no contraction in a fully contracted heart).

In clinical practice, cardiological evaluation comprises several observations, including the search for signs of cardiac dysfunction (dyspnoea or breathlessness) and the use of devices to evaluate blood flow and electrical characteristics. These observations may refer to individual heart cycles (such as in Echocardiographs), or series of cycles, named here “heart beating process”. The most commonly evaluated surrogates for the heart beating process are heart rate and pulse rhythm. “Heart rate” is commonly defined as the number of times a complete heart cycle event occurs within a given time, usually per minute. This measure is important to evaluate the response of the heart to body conditions, and evaluate the rhythmic functioning of the heart. It is called fast if the number of cycles is greater than normal, and slow if the number of cycles is smaller than normal. As the time frame of the measurement is rather arbitrary, we can also think of heart rates as describing the mean duration of cycles. “Pulse Rhythm” is commonly defined as the regularity between the time intervals in a set of three or more subsequent cycles. It is called rhythmic if the intervals between sequential cycles are similar; and arrhythmic if the intervals show great variation. Alterations of heart behaviour are seen in many diseases, and there are several names for most commonly observed patterns, like higher than normal (tachycardia) and slower than normal heart rate (bradycardia).

Some ontological models that lack any attributes for processes would treat heart rate as a regular observation, or a quality of the (human or animal) organism the heart is a part of, in the same fashion as body temperature. On a first sight this seems reasonable: at least in theory, it would be possible to calculate the heart rate based on complete knowledge of the chemical balance of the heart, the breathing cycle and some instantaneous measurement of heart contraction speed, in order to avoid the time-dependence. However, the situation becomes more complicated in cases in which we have to know the exact relation between some entity, like an event or a substance, and a resulting *change* in a heart rate. For example, a drug might cause a change in the duration of a heart cycle, directly affecting the heart rate. Therefore there should be a way to capture the knowledge of the effect that such a drug can have or to relate such a change back to substances which can cause it. This requires explicit representation of process attributes. We can also find situations in which process attribute changes is required to be recorded in medical records. For instance, the concept of heart rate variability, recently implicated in worse outcomes of cardiovascular diseases, is measured by the change of the heart rate in resting position and after heart rate decreasing-increasing manoeuvres (Valsalva) [13]. We are, therefore, interested in describing the process of change of the heart behaviour, finally identifying whether the patient’s heart is capable of varying its rhythm accordingly.

### Evaluation criteria

In order to produce objective results, we will use pre-defined criteria to evaluate the quality of the resulting representation artefact. Since we are dealing with a foundational issue, it is hard to create clear quantitative metrics for evaluation. However, by using a simplified model it is possible to state the main advantages and problems, and allow for future comparison with other methods. The competency questions^1^ to be answered by the model are:

 1. Given that we know the number and duration of *n* sequential heart cycles, can we categorise the heart beating process instance under the following classes:

 a. Normal heart rate for a 30 year-old;

 b. Fast heart rate for a 30 year-old.

 2. Given that the beating process is properly classified, can we provide representations that answer the following queries:

 a. Patient with bradycardia;

 b. Administered drugs that cause heart frequency increase;

 c. Query a triple store for diseases that co-occur with some arrhythmia.

 3. Can we represent the following medical statements (from real medical records):

 a. "Paroxysmal atrial fibrillation (diagnosis)";

 b. "Regular cardiac rhythm";

 c. "Sudden onset of palpitation";

 d. "History of supraventricular tachyarrhythmia";

 e. "No atrium-ventricular or intra-ventricular conduction abnormalities (ECG finding)";

 f. "Chronic atrial fibrillation (diagnosis)".

The answers will be qualitatively analysed with the following generic questions in mind, based on [14] :

 · Is it useful?

 · Does it produce the correct inferences?

 · How expressive is it relative to the alternatives, in particular to regular expressions?

 · Are there computationally more efficient solutions?

 · Which pattern should be chosen for a particular application?

### Implementation

For the implementation of the use case, an OWL DL ontology was created using Protégé 4 OWL Editor < version 4.1.0, Build = 239>. The ontology extends OGMS version of 2011-09-20, which is linked to BFO version 1.1 and the OBO Relation Ontology. Time-related information artefact classes were extracted from OBI through the MIREOT methodology [15].

The reasoners used were FaCT++ (version 1.5.3) and Hermit (version 1.3.5). They were run on an AMD A6-3410MX processor / 8 GB RAM computer, performing automatic classification of classes and their members, and probing for inconsistencies.

### Use case analysis

For the ontological analysis of the heart cycle, common medical expressions were collected during the execution of the Blood Project, a cooperation between the Ontology Research Group at the University at Buffalo and the Hemominas Foundation and the School of Information Science at the Federal University of Minas Gerais. Expressions were translated from Portuguese to English by a domain expert. Competence questions were developed loosely based on questions present in medical records (e.g. a diagnostic question concerning a drug as cause of bradycardia), and based on common functions present on Electronic Health Records (drug contra-indications). Use case requirements were broadly discussed between the authors, aiming to describe different aspects of medical reporting (patient state, disease progress and physician reasoning process).

## Results

Based on the statements contained in medical records, medical literature analysis and ontological analysis of the process, several key terms were identified. They were represented as OWL classes, building upon the OGMS [11].

### Design pattern for representation of process attributes of cyclical processes and implementation in OWL

In the most general sense: our pattern for representation requires the following:

 · That the overall process to which the attribute is being ascribed be composed of repeated sub-processes; and

 · That the repeated sub-processes be enumerated and have a duration.

**Figure 1:**
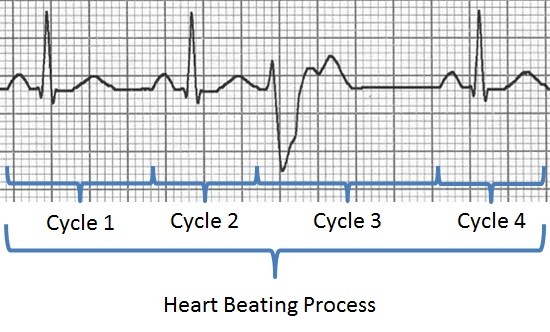
The heart beating process as represented in ECG.

**Figure 2:**
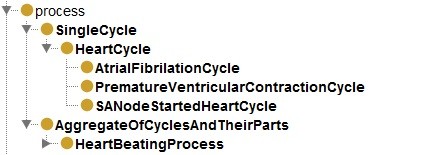
Cycle and Cyclic Process distinction.

The OWL model is built around the two classes: *heart beating process* and *heart cycle*. Whereas the former is a homomereous entity (there are parts of heart beating processes, which are, again heart beating processes) [16], the latter refers to the events that occur from the beginning of one heartbeat to the beginning of the next. A sequence of members of the class *heart_cycle* constitutes all members of the class *heart_beating_process*. If we cut a temporal region, which spans over a minute of this beating process we can count the number of full heart cycles that take place in that time period. The class *heart_cycle* has common properties to every cyclical process; and the heart beating process, which for any given period of time consists of a fiat collection of heart cycles. The generic classes were named “*single cycle”* and “*aggregate of cycles and their parts*”, respectively. This can be seen in Figure 1 and 2. We relate the cycles and the aggregate using the BioTop [17] relation “hasGranularPart”, since our goal is to relate collectives (aggregate of cycles) and the grains (the cycles themselves) that compose it [18].

**Figure 3:**
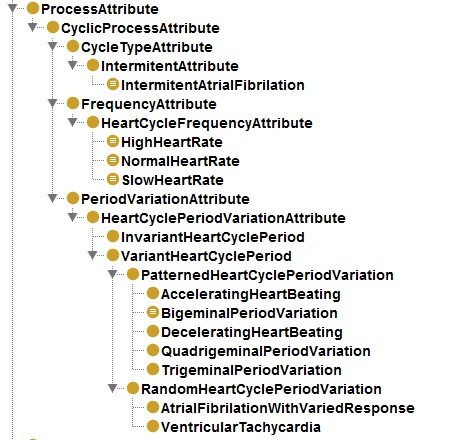
Process attributes of cyclic processes.

The second pattern was a distinction between structural dimensions of aggregate of cyclic processes. Following a corollary of the physical properties of cycles, we created three classes, according to frequency of the cycle (number of cycles in a given time), the variation between their periods (being the lack of significant variation called “*regular cycle*”) and the types of cycles that compose the aggregate of cycles. This can be seen in Figure 3.

To test the validity of these distinctions, OWL axioms were created, which are described below.

Our first axiomatisation attempt involved cardinality properties of the heart beating process. According to this view, every member of the class *heart_beating_process* is therefore the summation of individual cycles, members of the class *heart_cycle*.

(1) HeartBeatingProcesssubClassOf hasGranularPartmin2HeartCycle

(2) HeartBeatingProcesssubClassOfprocess

(3) HeartCyclesubClassOfprocess

(4) processsubClassOfexactly1hasDuration

The calculation of a heart rate, though at first sight straightforward, has several possible measurement procedures, particularly for detecting heart rate variation [13]. For the present purposes, we will consider that the measurement procedure does not alter the ontological description of a heart rate, namely, the description of how many individual heart cycles occurred in a process within a given time interval. Therefore, we defined a heart beating process according to its duration, following the convention put forward in [19]^2^. As mentioned before, this duration is arbitrary, but it follows common clinical practice and serves the purpose of unambiguously defining the correct scientific interpretation of heart rates.

(5) $$\begin{array}{l} \text{SixtySecondHeartBeatingProcess equivalent}\;\\ \;\text{ToHeartBeatingProcessand hasDuration}\\ \;\text{someSixtySecondTemporalInterval} \end{array}$$

OWL syntax allows us to express these counts by cardinality restrictions and pre-setting the duration of the analysed process, in order to allow classification. Normal heart rate is considered to be a beating process that contains between 60 to 100 individual cycles as parts. In this paper, we exclusively consider adult values for interpretation, due to clarity reasons, but it is important to note that additional information (the age of the paThe use of reasoners showed that such axiomatisation is practically infeasible, since the addition of this axiom increased classification time by more than 10 minutes. Both classifiers took very similar processing time in a quad-Core 8 GB RAM laptop computer. Even more importantly, the results of classification were never obtained – the classification process did not appear to terminate. Since the goal of this paper is not an evaluation of reasoners, this generic axiom was modified to use OWL data properties, maintaining the general modelling approach.

(6) $$\begin{array}{l} \text{HeartCycleFrequencyAttribute and}\\ \;(\text{is}\mathrm{Pr}\text{ocessAttributeOf}\;\text{some}\\ \;(\text{SixtySecondHeartBeatingProcess}\;\text{and}\\ \;(\text{hasNumberOfGrains}\;\text{some int}\;\left[\geq 60,\leq 100\right] \end{array}$$

Here, classification time was reduced; the time taken with the cardinality constraint was in the order of 1,892,256 ms (Fact++, but the results of both reasoners were comparable), but only 12,967 ms for the data property processing. While this alternative representation of cardinality using data properties was successful in properly classifying heart rates, a different approach was required for representing acceleration and complex arrhythmic patterns. The term “pattern” is used in the ontology to represent a common, recurrent and clinically important combination of entities that is used to describe a part or the whole process. An acceleration pattern could be translated as “an attribute of a collection of sequential cycles, in which every cycle, except the first, has a shorter duration than the one that immediately precedes it” (more tolerant and realistic variations would allow a certain amount of exceptions). But already this most simple definition proved impossible to be represented in OWL, since it requires keeping track of individuals across a variable number of cycles. Therefore, we represented rhythm patterns as primitive classes.

### Evaluation

According to the pre-defined competency questions, we obtained the following results, addressing the above mentioned:

As can be seen in Table 1, most statements can be successfully expressed by describing the individual cycles and the number of cycles in a given time frame, making the language statements logically sound and internally coherent. However, some classes that refer to acceleration could only be formalised in first-order logic, which is further explored in the next section.

**Table 1:** Heart rate representation issues and their ontological description

**Problem**	**Representation**	**Comments**
1.a. Normal heart rate for a 30 year-old	*HeartCycleFrequencyAttribute* and **(isProcessAttributeOf** only (*SixtySecondHeartBeatingProcess* and (**hasPart** min 60 *HeartCycle*) and (**hasPart** max 100 *HeartCycle*)))	The model allowed proper representation and automatic classification of those classes. Such representation can be done through fully defined classes – therefore precluding the need of process attributes
1.b. Fast heart rate for a 30 year-old	*HeartCycleFrequencyAttribute* and (**isProcessAttributeOf** some (*SixtySecondHeartBeatingProcess* and (**hasNumberOfGrains** only int[> 100])))	
2.a. Patient with bradycardia	*BradycardiacPatient***equivalentTo***Human* and (**hasPart** some (*Heart* and **participatesIn** some (*HeartBeatingProcess* and **hasProcessAttribute** some *SlowHeartRate*)))	
2.b. Administered drugs that cause heart frequency increase	*TachycardicEffect***equivalentTo***DrugFunction* and (**inheresIn** some (*Drug* and **participatesIn** some (*TherapeuticMedicationAdministration* and **precedes** some (*HeartBeatingProcess* and **hasProcessAttribute** some *AcceleratingHeartBeating*))))	
2.c. Query a triple store for diseases that co-occur with some arrhythmia	SELECT ?Disease WHERE ?*ParticularDisease***isInstanceOf** ?*Disease*.?*ParticularDisease***inheresIn** ?*Human*.?*Human***hasPart** ?*Heart*. ?*Heart***participatesIn** ?*HeartBeatingProcess*. ?*HeartBeatingProcess***hasProcessAttribute** ?*ProcessAttribute*. ?*ProcessAttribute***isInstanceOf***ArrhithmicHeartRate*.	
3.a. "Paroxysmal Atrial Fibrillation (diagnosis)"	*AtrialFibrilationHeartBeating***equivalentTo***HeartBeatingProcess* and (**hasPart** only *AtrialFibrilationCycle*) and **hasNumberOfGrains** some integer [>1] *SANodeHeartBeating***equivalentTo***HeartBeatingProcess* and (**hasPart** only *SANodeStartedHeartCycle*) and **hasNumberOfGrains** some integer [>1] *IntermitentAtrialFibrilationAttribute***equivalentTo isProcessAttributeOf** some (*HeartBeatingProcess* and (**hasPart** min 2 *AtrialFibrilationHeartBeating*) and (**hasPart** some *SANodeHeartBeating*))	Atrial fibrillation can be modelled as a collection of atrial fibrillation cycles, in which the atrial contraction is not regulated by the SA node. The paroxysmal can be modelled as a process attribute of processes that have as part more than 1 instance of the same type of process.
3.b. "Regular cardiac rhythm"	(∀x)(∀y)(∀z) (HeartBeating(x) Λ HeartCycle(y) Λ HeartCycle(z) Λ has_attribute(x, Invariant_heart_cycle_period) Λ (y ≠ z) Λ partOf(y,x) Λ partOf(z,x)→ durationOf(y) = durationOf(z))	Fully defined in FOL by stating that the duration of each cycle part of the heart beating has the same duration as any other cycle part of the heart beating.
3.c. "sudden onset of palpitation"	**hasProcessAttribute** some (*NormalHeartRate* and **precedes** some *AcceleratingHeartBeating*)	Palpitation will require some primitive as acceleration has been shown as difficult to represent in OWL. However, the “sudden” can be expressed as a process attribute “normal heart rate” that precedes an acceleration attribute, and the time instant of the end boundary of the former is the same as the time instant of the initial boundary of the latter.
3.d. "History of supraventricular tachyarrhythmia"	**isAbout** some *HeartBeatingProcess* and (**hasProcessAttribute** some *HighHeartRate*) and (**hasPart** only *SANodeStartedHeartCycle*)	History can be expressed reusing IAO relation “is About”.
3.e. "no atrium-ventricular or intra-ventricular conduction abnormalities (EKG finding)"	**isAbout** some *HeartBeatingProcess* and (**hasProcessAttribute** only *NormalHeartRate*) and (**hasProcessAttribute** only *InvariantHeartCyclePeriod*) and (**hasPart** only *SANodeStartedHeartCycle*)	See 3.b and 3.d.
3.f. "chronic atrial fibrillation (diagnosis)"	**isAbout** some *ChronicAtrialFibrilationAttribute ChronicAtrialFibrilationAttribute ***equivalentTo isProcessAttributeOf** some (*HeartBeatingProcess* and (**hasPart** only *AtrialFibrilationCycle*))	Similar to 3.a, but in this case the beating has only atrial fibrillation cycles as part.

### Formalisation of process patterns

We made an attempt to detail some points relating to a first-order logic (FOL) representation of the above process patterns, bearing in mind that most bio-ontologies are currently formalised in OWL or the OBO language and therefore would not be able to directly make use of such a formalisation. Emerging tools for the integration of ontology modules specified in different logical frameworks could provide a pragmatic solution to this problem in the near future [20].

A process q is constituted by a sequence of part processes P = {p_1_, p_2_, …p_n_ }

All processes x have a duration dur (x) which is denoted by a real number in some time scale

(7) $$\;\wedge_{k=1}^{n}\text{hasPart (q, pk})\wedge_{k=2}^{n}\text{follows (pk, pk}-1)$$

Ideal accelerating process: follows (p_k_, p_k-1_) → duration(p_k_) < duration(p_k-1_)

Ideal decelerating process: follows (p_k_, p_k-1_) → duration(p_k_) > duration(p_k-1_)

Ideal even process: follows (p_k_, p_k-1_) → duration(p_k_) = duration(p_k-1_)

How much real rhythmic processes are to be classified requires a fiat division of the continuum between the ac- (de-)celerating and even processes. Considering the numerical representation of the durations of each individual cycle, (5, 5, 4, 4, 5, 3, 3, 2, 2, 2, 3, 2, 1, 2, 1, 1) would be closer to an accelerating process than to an even process.

## Discussion

While some authors argue that ontological realism should be relaxed in some aspects of ontology modelling [21] due to its apparent over-complexity, the creation of ad-hoc new primitive classes has unforeseeable downstream consequences. One of the main benefits of the realist approach is to allow modelling convergence despite domain-specific and application-specific perspective differences by using scientific results and the interdisciplinary bridging perspective of philosophical ontology as a methodology to arrive at more precise and unambiguous ontological structure as a substitute for unexamined natural language assertions such as form the strategy behind terminological resources such as MeSH. Representations of the world according to the consensual scientific discourse guarantee reliable and robust representation artefacts [22]. Our description of process attributes maintains this principle, but at the same time provides sufficient expressivity to meet the domain requirements.

**Figure 4:**
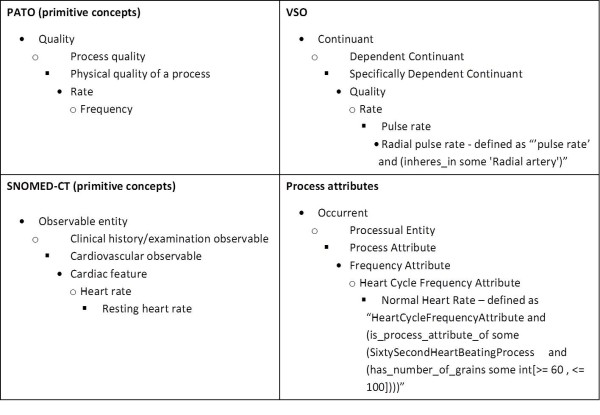
Comparison between different process qualities representations.

When analysed more deeply, process attributes as used in domain terminologies reveal themselves to be a loosely related set of descriptions, reifications and analogies used to communicate some characteristics of events in natural language. We provide in Figure 4 a comparison between SNOMED, PATO, VSO and the present approach.

The most basic characteristic of the heart cycle is the fact that it is a cycle and can be described according to its frequency and variation of sequential periods. A frequency of a cycle refers to its cardinality within some given time which can itself be fully expressed using primitive ontological constructs. Likewise, the duration of periods are real entities and require no special construct. Time intervals are occurrents, just as processes, and therefore not qualities. Durations of time intervals are numeric values.

Despite the underlying compatibility with primitive constructs, a full definition of attributes in our use case was possible only in some cases due to limitations in the underlying logical description language. The right degree of expressivity of a logical language has been subject to very long dispute. However, due to its balance between expressivity, decidability, and most importantly, due to its being an official W3C recommendation, the Web Ontology Language (OWL-DL) is the *de facto* standard for representing ontologies across many domains including that of biomedicine. OWL is based on description logics, which are carefully selected decidable subsets of first order logic. Our main difficulty in defining some attributes (such as acceleration of heart rate) results from the lack of generalised support for arithmetical relations on individuals, such as *greater than* and *less than*, which are needed in our acceleration examples between instances of ordered cycles. Therefore, we could not assert that *cycle n* immediately precedes *cycle n + 1* and has a greater duration than *cycle n + 1*.

While not completely representable in OWL, we argue that the FOL definition we provided for some process attributes still contributes to restricting possible interpretations of the class. We can, for instance, distinguish between an strictly accelerating heart beating (every cycle is longer than the one it precedes), a constant accelerating cycle (every cycle is longer than the one it precedes, being the difference between any 2 sequential cycles duration always the same) and an accelerated heart beating, or palpitation (as defined in 3.c.i). The distinctions were shown to be quite adequate for representing the general meaning of common expressions and categories for cardiac arrhythmias, when tested against the criteria. It is important to emphasise, however, that natural language use of these words is much more relaxed and context-dependent, which may require local adaptations according to application needs. Also, many distinctions require the proper identification and classification of types of cycle, which is a rich subject area of its own and out of scope for the current work. Finally, the FOL definition cannot be used for reasoning purposes, since consistency checking is done by OWL reasoners (classification and consistency checking). While our representation could be extended to more expressive logics, this would raise additional concerns regarding decidability that are not in the scope of this paper.

A special case is the description of rhythm patterns like the bigeminal^3^ rhythm. While at first classified under patterned period variation – since its rhythm is easily recognisable, with a short cycle followed by a long cycle followed by a short cycle, and so on – it can also be described according to the origin of the electrical impulse leading to heart contraction (supra-ventricular and ventricular).

### Application to non-cyclical use cases

It is important to highlight that the mentioned design patterns apply exclusively to cyclic processes. However, our approach was developed for generically representing attributes aside from cyclic process. In several cases, we have to decompose a complex process in order to understand what an attribute intends to describe. For example, a pain process can be understood as the summation of nociceptor stimuli. Here, however, not the duration of the action potential matters but the frequency with which action potentials are produced by a group of nociceptors. Allowing the exact description of the process does not mean that such a precise measurement is possible in clinical practice. As discussed in [23], separation between the fact and information about the fact can be used to properly describe this situation (using the OWL ‘only’ operator). Also, many pain-related entities common to clinical practice are epistemological entities, which must be carefully evaluated for suitability in realist ontologies [24].

Pregnancy is another highly complex process, due to its mutually coordinated structural and functional changes in (at least) two organisms. The pregnancy process, focusing on the mother’s organism is commonly dissected by fiat into three trimesters, whereas the development of the offspring is split into embryogenesis and foetal development. The sub-process that terminates the pregnancy is the delivery, which again, can be split into a series of processes, such as the sequence of configuration of the baby’s head and body within the birth channel, and the progress of the mother’s labour. The variants of the pregnancy process are manifold in terms of

 · Duration

 of the whole process, or process parts, such as labour of repetitive phases such as uterine contractions in relation to the intermittent latency phases

 · count

 number of contractions

 · intensity

 pain, contractions

 · extra process parts

 surgical interventions such as episiotomy or caesarean section

 complications of pregnancy such as eclampsia or diabetes

 · missing process parts

 failure of descent of the foetal head

 embryonic defects

The pregnancy is also characterised by its participants (mother, offspring), their related body parts and qualities, such as number of offspring, their size, missing or supernumerary parts etc.

Due to the myriad of determinants of a pregnancy process, a classification into “normal” and “abnormal” cannot be reduced to hard criteria. Apart from some extreme situations (e.g. foetal death, miscarriage), the boundary between the normal and the abnormal is fuzzy, as is common in medicine. We argue that the correct description of participants and sub-processes allow proper comparison of different abnormalities, without the arbitrary creation of different terms. Our approach promotes the precise description of each occurrent and participant of the pregnancy process, in order to maintain modelling coherence and accurate representation. For instance, premature labour could be defined according to the time span between each contraction cycle or the cardinality of contraction within a given time span, and the occurrence of these contractions within the time interval spanning from conception to the 37^th^ week after conception. Therefore, it is clear what makes normal labour and premature labour pregnancies similar and what is the distinction between the normal and pathological process parts.

As pointed out by the heart cycle example, the logical language (in this case OWL-DL) imposes limits on what can be adequately represented therein. The proper evaluation of this limitation in ontological representation and reasoning remains to be evaluated. However, it is important to point out that ontological analysis here proposed is independent of particular representations, and is coherent with the philosophical view put forward in BFO foundational papers. Particularly, this approach is coherent with the view that “processes do not change, because processes are changes” put forward by Smith [25]. It is also compatible with BFO 2.0, which introduces *Process profile* as a special sort of processual parts [19]. In this paper, we do not propose a different interpretation, but have rather outlined a complementary approach than a proper ontological definition of complex processes. Therefore, instead of determining profiles according to an *ad hoc* structural dimension of a process, process attributes require a precise definition in terms of the kinds of participants, participant qualities and sub-processes that characterize the (attributed) process.

## Conclusions

Attributes of process – or process qualities – are common descriptions within most communities. While BFO 2.0 is now introducing a new category to fill this gap, modelling restrictions are still required to promote interoperability. Strict adherence to engineering guidelines and best practices of logical representation ensures that the resulting ontology will be adequate to the domain and useful for specific applications. We have shown that processes qualities can be successfully represented by the duration of the whole process, its parts (sub-processes), their participants, and the qualities of the participants. One could therefore argue that process qualities are not justified as first-class citizens in biomedical ontologies. They should rather be included for convenience as fully defined classes. However, as their full definition often requires logical machinery that exceeds the capabilities of current reasoning devices, and may adversely impact reasoning performance even in cases where the expressivity is supported, there may be a pragmatic need to accommodate them as primitives.

By describing in detail the application of this pattern to heart rate modelling, we have shown the suitability of cardinality, distinction of parts of process and cycle properties to define process attributes. Further work is required to refine these patterns of representation and increase coverage of the approach, while maintaining logical and philosophical consistency.

## Endnotes

^1^Some definitions of medical terms used to formulate competency questions:

Paroxysmal atrial fibrillation: Atrial fibrillation that occur in episodes, separated by periods of normal heart beating

Palpitation: Sudden increase in heart rate

Tachyarrhythmia: Cardiac rhythm disorder in which the heart rate is abnormally high

^2^process *p* has duration *d* : process *p* occupies temporal region *t* and *t* instance_of universal *temporal region with duration d*

^3^Bigeminal rhythm: Heart beating characterized by a normal sinusal beat succeeded by a premature beat – therefore, the beats occur in pairs, showing a particular rhythm.

## Competing interests

The authors declare that they have no competing interests.

## Authors' contributions

AQA developed the ontology and drafted the manuscript. JH and WB provided substantial input to the ontology development and to the manuscript, and created the FOL and SPARQL representations. SS conceived of the study, and participated in its design and coordination and helped to draft the manuscript. All authors read and approved the final manuscript.
